# Tetramethylpyrazine-Inducible Promoter Region from *Rhodococcus jostii* TMP1

**DOI:** 10.3390/molecules23071530

**Published:** 2018-06-25

**Authors:** Rūta Stanislauskienė, Simonas Kutanovas, Laura Kalinienė, Maksim Bratchikov, Rolandas Meškys

**Affiliations:** 1 Department of Molecular Microbiology and Biotechnology, Institute of Biochemistry, Life Sciences Center, Vilnius University, Saulėtekio al. 7, LT-10257 Vilnius, Lithuania; s.k.pastas@gmail.com (S.K.); laura.kaliniene@bchi.vu.lt (L.K.); rolandas.meskys@bchi.vu.lt (R.M.); 2 Department of Physiology, Biochemistry, Microbiology and Laboratory Medicine, Faculty of Medicine, Vilnius University, M. K. Čiurlionio 21, LT-03101 Vilnius, Lithuania; maksim@biosta.lt

**Keywords:** *Rhodococcus* sp., inducible promoter, 2,3,5,6-tetramethylpyrazine, LAL subfamily, LuxR

## Abstract

An inducible promoter region, P_TTMP_ (tetramethylpyrazine [TTMP]), has been identified upstream of the *tpdABC* operon, which contains the genes required for the initial degradation of 2,3,5,6-tetramethylpyrazine in *Rhodococcus jostii* TMP1 bacteria. In this work, the promoter region was fused with the gene for the enhanced green fluorescent protein (EGFP) to investigate the activity of P_TTMP_ by measuring the fluorescence of bacteria. The highest promoter activity was observed when bacteria were grown in a nutrient broth (NB) medium supplemented with 5 mM 2,3,5,6-tetramethylpyrazine for 48 h. Using a primer extension reaction, two transcriptional start sites for *tpdA* were identified, and the putative −35 and −10 promoter motifs were determined. The minimal promoter along with two 15 bp long direct repeats and two 7 bp inverted sequences were identified. Also, the influence of the promoter elements on the activity of P_TTMP_ were determined using site-directed mutagenesis. Furthermore, P_TTMP_ was shown to be induced by pyrazine derivatives containing methyl groups in the 2- and 5-positions of the heterocyclic ring, in the presence of the LuxR family transcriptional activator TpdR.

## 1. Introduction

*Rhodococcus* spp. are gram-positive, high GC-content bacteria that are found in many environmental niches, namely: tropical, arctic, and arid soils, as well as in marine and deep-sea sediments [1]. Rhodococci are able to degrade or metabolically transform both short- and long-chain hydrocarbons as well as aromatic, heteroaromatic, and polycyclic aromatic compounds, and use them as the sole carbon and energy source. Their metabolic versatility depends on cell physiology, adjustment to new substrates, and the ability to gain and retain a wide range of catabolic genes [2]. The accomplishment of every catabolic pathway basically depends on two elements, the catabolic enzymes leading to mineralization of the compound and the regulatory elements. The regulatory proteins and regulated promoters are the major elements to control the transcription of catabolic genes and to ensure an adequate metabolic return to the specific substrate that serves as the nutrient source [3].

It has become increasingly evident that rhodococci play a leading role in the biodegradation of a remarkable variety of compounds, some of which are highly toxic to many other bacterial species [1]. Therefore, it is unsurprising that the number of studies on the rhodococci metabolism as well as those on the regulation of catabolic pathways in these bacteria has been growing rapidly. More than 10 years ago, it was shown that *Rhodococcus* is the predominant alkane degrader [1]. Later, the inducible promoters of *alkB* genes were described for n-alkane-degrading *Rhodococcus ruber* SP2B and *Rhodococcus* sp. BCP1 bacteria [4,5]. Through molecular analysis as well as genomic and biochemical studies, it was determined that rhodococci are also capable of degrading aromatic and aliphatic compounds. For example, *R. jostii* strain RHA1 can utilize phenol whereas *R. erythropolis* CCM2595 can utilize both phenol and catechol. In the case of both *Rhodococcus* strains, the catabolic genes and their promoters have been analyzed by Vesely et al. [6] and Szőköl et al. [7], respectively. Some *Rhodococcus* spp. strains are capable of utilizing complex compounds, such as aromatic hydrocarbons containing two benzene rings or heterocyclic compounds with two benzene rings fused to a central 5-membered ring (dibenzothiophene, dibenzofuran). Five transcriptional promoters of biphenyl degradation genes that are under the control of *bph*ST-coding two-component regulatory system have been characterized in *Rhodococcus* strain RHA1 [8]. The promoters of genes involved in the degradation of dibenzothiophene, dibenzofuran, have been described as well [9].

Several expression vectors based on the replicons and promoters of rhodococci have been constructed recently. The promoter of the thiostrepton-regulated gene from the *Rhodococcus opacus* strain DSM 44193 [10] has been used to create the inducible expression vectors (pTip) that have been successfully applied in several *Rhodococcus* species [11]. In 2012, using the upstream region of isocitrate lyase from *Rhodococcus erythropolis* PR4, a number of novel methanol-inducible and strong constitutive expression vectors were constructed by Kagawa and colleagues [12]. To investigate the cellulose degradation in *Rhodococcus opacus* PD630, the shuttle vectors pEC-K18mob2 and pJAM2 containing *lac* [13] and acetamidase *ace* [14] promoters, respectively, have been used for the cloning of six different cellulase genes. It has been revealed that both promoters are constitutively expressed in the aforementioned bacteria [15].

The catabolic operons involved in the biodegradation of xenobiotics in *Rhodococcus* spp. also hold promise for synthetic biology. On the basis of the pSRKBB-empty plasmid containing the lac promoter, a BioBrick^TM^ compatible plasmid system for *Rhodococcus* spp. has been constructed [16]. Such an expression system makes rhodococci suitable for use as a biocatalyst or as a host for bioproduction. Moreover, transcription regulator-based inducible systems , which are widely spread in microorganisms to coordinate metabolic pathways, have been recently adapted as genetically-encoded biosensors, which respond to a variety of compounds. Such systems are used to screen for metabolically engineered microbial strains as well as novel biocatalysts [17,18,19,20,21,22,23]. To expand the availability of sensors with appropriate characteristics, more studies aimed at identifying novel transcription regulators that are applicable for biosensor design in different expression hosts are required.

Recently, the degradation pathway of 2,3,5,6-tetramethylpyrazine (TTMP) in *Rhodococcus jostii* TMP1 bacteria has been elucidated. The *tpdA-tpdE* operon-containing genes required for the initial degradation of TTMP as well as the upstream region of the *tpdA* gene harbouring a putative promoter (P_TTMP_), which is specifically activated by TTMP, have been identified [24]. In this study, we present data on the structure and regulation of this TTMP-inducible promoter.

## 2. Results

### 2.1. Analysis of the Promoter Activity in Rhodococcus josti TMP1 Cells

**Figure 1:**
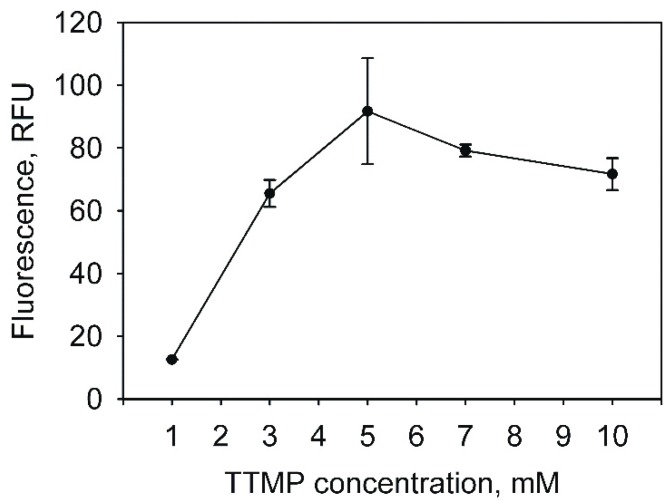
The dependence of the enhanced green fluorescent protein (EGFP) expression in *Rhodococcus jostii* TMP1 on the concentration of tetramethylpyrazine (TTMP). The EGFP fluorescence was measured by a plate reader (λ_ex_ = 485 nm; λ_em_ = 510 nm); the data are presented as averages of triplicate measurements with error bars.

For the initial characterization of the promoter P_TTMP_, the plasmid pART3-5′UTR-gfp was obtained by fusing the PCR-amplified 277 bp fragment of the upstream region of *tpdA* [24] with the enhanced green fluorescent protein (EGFP) gene from the pART3-gfp plasmid. The *R. jostii* TMP1 cells were then transformed with the constructed recombinant plasmid. To determine the optimal concentration of TTMP for the highest promoter activity, the cells harbouring pART3-5′UTR-gfp were cultivated for 48 h in 20 mL of a nutrient broth (NB) medium supplemented with different TTMP concentrations. The expression level from the promoter P_TTMP_ was monitored measuring the intensity of the fluorescence of the bacterial cultures. The highest promoter activity was observed when the medium contained 5 mM TTMP, but even 1 mM of TTMP induced the expression of EGFP (Figure 1).

The time-course of the promoter activity was analysed by cultivating *R. jostii* TMP1 cells harbouring pART3-5′UTR-gfp in NB, EFA, and minimal media, supplemented with 5 mM TTMP. The synthesis of EGFP gradually increased, reaching the highest expression level at the 44th hour of cultivation in the NB medium. When the NB was replaced with EFA, the maximum production of EGFP was observed after 24 h of incubation, however, the registered fluorescence values were almost 6-fold lower than those obtained using the NB medium. Furthermore, an even lower level of fluorescence (approximately 16-fold lower than that registered using the NB) was observed when the cells were cultivated in a minimal medium (data not shown).

Aside from TTMP, *R. jostii* TMP1 is also capable of using pyridine as a sole carbon and energy source [25]. Thus, both glucose and pyridine were used to investigate the possible repression or induction of transcription from the promoter P_TTMP_. The cells harbouring thepART3-5′UTR-gfp were grown in an EFA medium, supplemented with the aforementioned compounds at different concentrations (0.05, 0.1, 0.5, and 1.0%) and 5 mM TTMP. At concentrations of 0.05 and 0.1%, neither the glucose nor pyridine diminished the transcription from the P_TTMP_ promoter. However, in the presence of TTMP, both 0.5 and 1.0% of the pyridine inhibited the growth of bacteria.

### 2.2. Analysis of the Minimal Promoter Sequence and Transcription Start Sites

**Figure 2:**
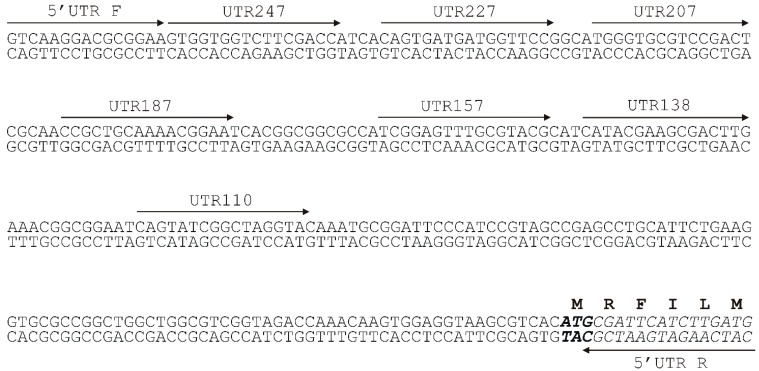
A scheme for the determination of a minimal promoter sequence. The primers used for the amplification of the upstream region of the *tpdA* gene are marked with arrows. The amplified DNA fragments were fused to the *egfp* gene in pART3-gfp.

**Figure 3:**
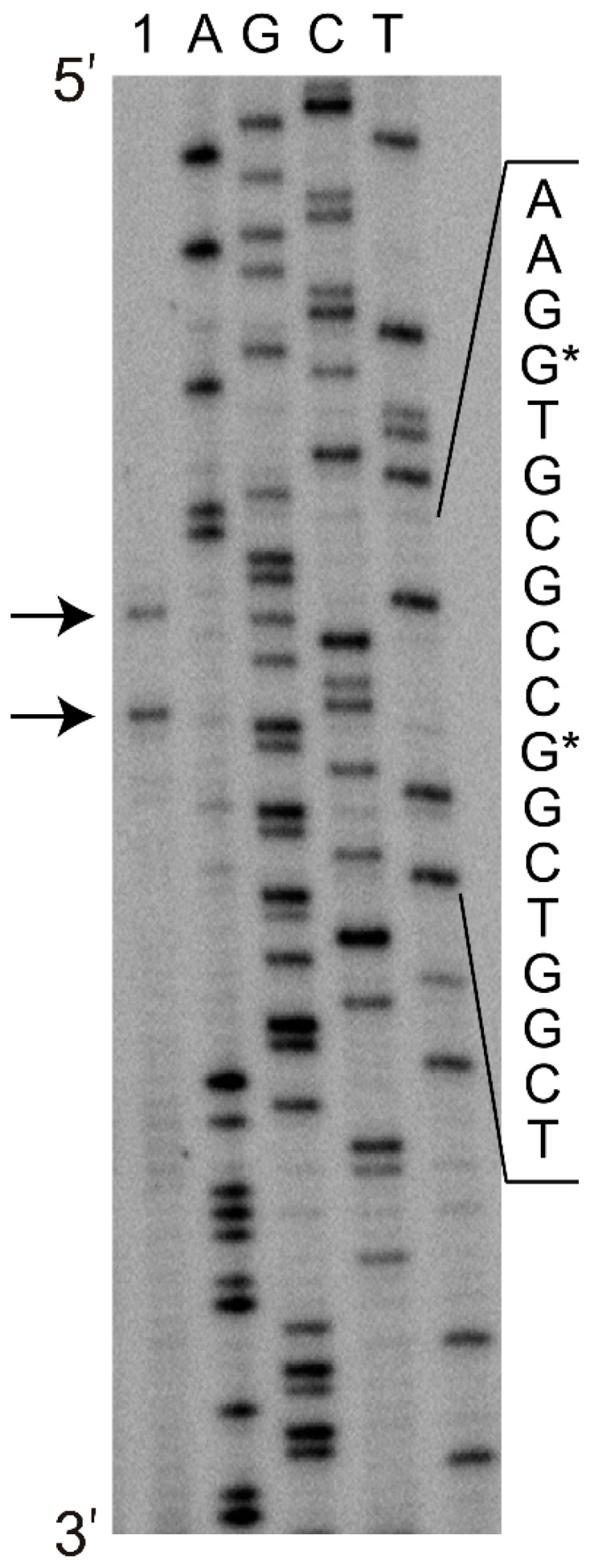
Determination of the transcription start site by primer extension analysis using the total RNA from *R. jostii* TMP1 harbouring the pART3-5′UTR-gfp plasmid. The extended product was analysed alongside a DNA sequencing reaction, using the same primer. The transcription initiation sites are indicated by arrows, and the corresponding nucleotides in the DNA sequence are marked by asterisks.

**Figure 4:**
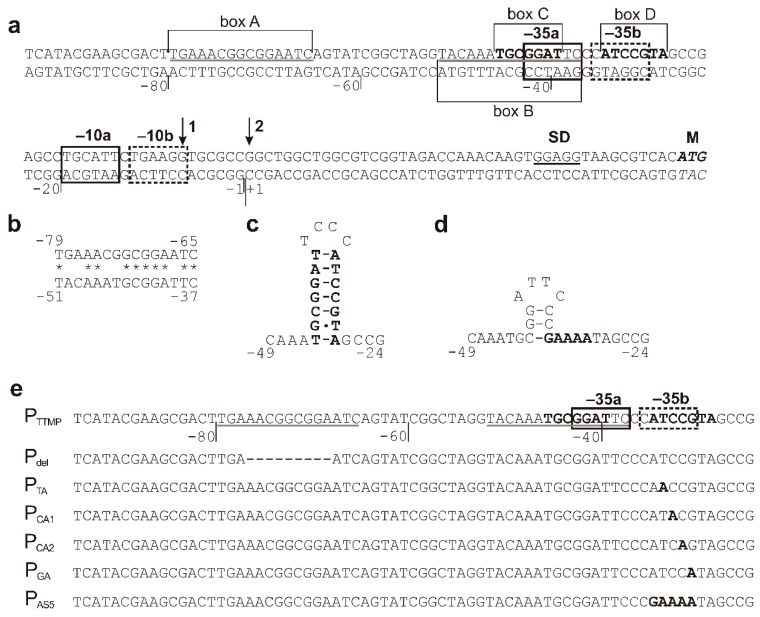
(**a**) P_TTMP_ core promoter elements. Arrows indicate transcription start sites, the −10 and −35 regions are boxed (the elements of the first promoter are indicated by solid-line boxes and the elements of the second promoter are indicated by dotted-line boxes), and the nucleotides that potentially form the secondary structures are given in bold; nucleotide sequence repeats are underlined by a grey line; Shine–Dalgarno sequence is underlined by a black line; the first translation codon is marked. (**b**) The comparison of 15 nt direct repeats. (**c**) The predicted secondary structure formed by the 7 nt inverted sequences. (**d**) A new secondary structure formed after the mutation of the promoter sequence (changed nucleotides are given in bold). (**e**) The mutations of P_TTMP_. The top line represents the wild-type promoter sequence. The dash indicates the deleted nucleotide; the substituted nucleotides are given in bold. Boxed regions correspond to the −35 promoter elements; nucleotides that potentially form the secondary structures are given in bold; and the nucleotide sequence repeats are underlined by a grey line.

To determine the minimal promoter sequence, the regions upstream of the *tpdA* gene were amplified (Figure 2) using the primers mentioned in Materials and Methods. The resulting PCR-generated fragments of different lengths were then individually fused with the gene for EGFP from the pART3-gfp vector. Notably, in the presence of TTMP, only the cells transformed with the recombinant plasmid, which carried the shortest 138 nt PCR product, failed to synthesise the EGFP. The transcription start site was determined by a primer extension analysis (detailed in Materials and Methods). Using the total RNA extracted from *R. jostii* TMP1 harbouring the pART3-5′UTR-gfp plasmid, two transcription start sites, 45 and 52 nt upstream of the translation initiation codon of *tpdA*, were detected (Figure 3), which hinted at the presence of two putative promoters (Figure 4).

Two 15 nt repeats (box A, −79 to −65; and B, −51 to −37) (Figure 4b) with 13 nt spacing, and two short 7 nt inverted repeats (box C, −45 to −39; and D, −34 to −28) were detected by visual inspection of the P_TTMP_ promoter sequences. The short inverted repeats, C and D, that could potentially form a hairpin structure (Figure 4c) were separated by four nucleotides. The direct repeat B completely overlapped the promoter −35a region, while the −35b region overlapped the inverted repeat D.

**Figure 5:**
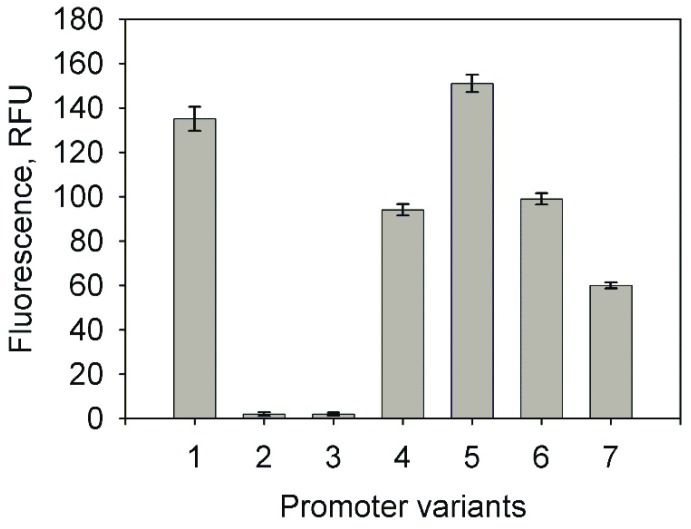
Effect of the promoter sequence mutations on the expression of EGFP in *Rhodococcus jostii* TMP1. 1—wild-type P_TTMP_ sequence; 2—P_del_; 3—P_TA_; 4—P_CA1_; 5—P_CA2_; 6—P_GA_, 7—P_AS5_. The effect of mutations on the EGFP expression was investigated by determining the intensity of the bacterial fluorescence. Bacteria were grown in the nutrient broth (NB) medium, containing 5 mM TTMP for 48 h. The fluorescence was measured by a plate reader (λ_ex_ = 485 nm; λ_em_ = 510 nm); the data are presented as the averages of the triplicate measurements with error bars.

To investigate the role of the A, B, C, and D motifs on P_TTMP_ activity, a number of mutations were introduced into the promoter sequence, as described in Materials and Methods. The individual mutated DNA fragments were cloned into pART3-gfp to obtain EGFP fusion constructs. A nine-nucleotide deletion was introduced into the box A, potentially disrupting its interaction with the sequence of the B box. Also, five box D mutants were constructed (Figure 4e) with the aim to prevent the formation of the putative secondary structure, depicted in Figure 4c. The effect of mutations on the promoter activity was investigated by measuring the intensity of the EGFP fluorescence in bacteria. A nine-nucleotide deletion in region A, and a single point mutation of T to A at position −33 in the box D, had the greatest negative effect on promoter activity. In both cases, the fluorescence was undetectable, suggesting that the EGFP gene transcription was not induced. The replacement of either C (−32) or G (−30) by adenine in the region D led to a slight reduction in the EGFP expression. When cytosine was replaced by adenine at position −31, the promoter activity increased compared with that of the wild-type P_TTMP_ (Figure 5). A five-nucleotide deletion in the D box did not abolish the promoter activity, however, the cells showed two-fold lower EGFP levels as compared with the *R. jostii* cells carrying plasmids with wild-type P_TTMP_. Notably, while the putative hairpin structure, depicted in Figure 4c, was disrupted by the aforementioned deletion, an alternative secondary structure could have formed (Figure 4d).

### 2.3. Analysis of the TpdR Regulator

**Figure 6:**
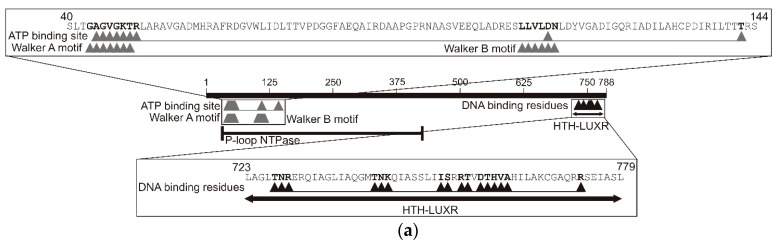

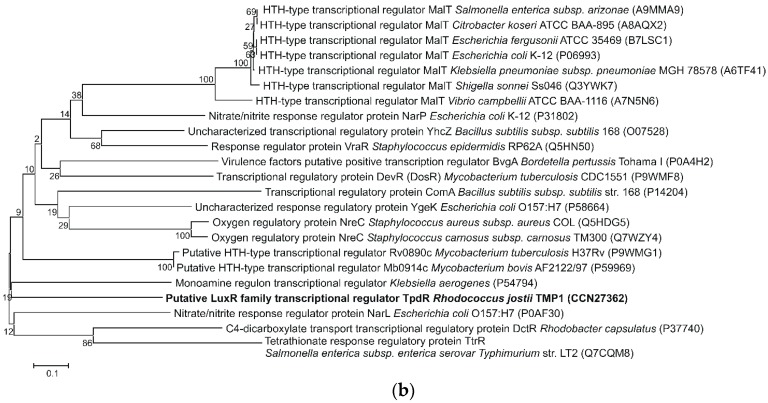
(**a**) Domains and motifs identified in the TpdR protein. (**b**) Neighbour-joining tree based on the alignment of the amino acid sequences of the C-terminal HTH LuxR domain of TpdR protein from *Rhodococcus jostii* TMP1 and other functionally diverse proteins from different bacteria. The percentage of replicate trees in which the associated taxa clustered together in the bootstrap test (500 replicates) are shown next to the branches. The evolutionary distances were computed using the Poisson correction method. All positions containing gaps and missing data were eliminated from the dataset using the complete deletion option. The scale bar represents the expected amino acid substitutions per position. The GenBank accession number is indicated for each protein. The phylogenetic analysis was performed using MEGA 5.0 [27].

Since the degradation of TTMP in *R. jostii* TMP1 bacteria is an inducible process, it was hypothesised that the protein encoded by *tpdR* may be the regulator of the *tpdA* expression, and likely acts as an activator rather than as a repressor. The bioinformatic analysis of the *tpdR* gene revealed that it contained 2364 nt and encoded a protein of 788 amino acids with a predicted molecular mass of 86 kDa. The analysis of the deduced amino acid sequence of TpdR showed that the protein belonged to the transcription regulators of the LuxR family [24]. The LuxR helix-turn-helix (HTH) DNA-binding domain was determined to be in the C-terminus of the protein. In the N terminus of TpdR, the typical nucleotide triphosphate (NTP)-binding domain along with the Walker A (43‒50 a. a.) and Walker B (109‒114 a. a.) motifs were identified as well (Figure 6a). These features suggested that TpdR may be a member of the large ATP-binding LuxR (LAL) subfamily of bacterial regulators [26].

Using the BLAST algorithm (https://blast.ncbi.nlm.nih.gov/Blast.cgi) of UniprotKB/Swissprot database, it was determined that TpdR shares homology with the LuxR-type HTH domain-containing response regulators (transcriptional activators or repressors, the length of proteins varied from 197 to 902 amino acids) from several genera, including *Mycobacterium*, *Staphylococcus*, *Bacillus*, *Escherichia*, and *Bordetella*. The phylogenetic analysis based on the alignment of the amino acid sequences of TpdR and other LuxR-type homologous proteins revealed that the protein from *R. jostii* TMP1 was phylogenetically distinct from other regulators, since the grouping with a single protein, the positive regulator of the monoamine regulon in *Klebsiella aerogenes*, was statistically reliable (Figure 6b).

To investigate the role of TpdR in the regulation of P_TTMP_, a pART3-5′UTR-gfp plasmid was transformed into *R. erythropolis* SQ1 cells. Although the bacteria were grown on a TTMP-containing medium, EGFP fluorescence was not observed. Subsequently, *tpdR* and its upstream region was amplified by PCR, using Reg_F_Xba and Reg_R_Xba primers, and the obtained fragment was cloned into the pART3-5′UTR-gfp downstream EGFP gene. The recombinant pART3-5′UTR-gfp-R plasmid was used to transform *R. erythropolis* SQ1 cells that were then grown on a nutrient agar (NA) medium, supplemented with 0.05% of TTMP or without an inducer. The EGFP fluorescence was observed in the *R. erythropolis* SQ1 cultivated with TTMP only. This result confirmed that TpdR regulated the expression from P_TTMP_ and was a transcriptional activator.

**Figure 7:**
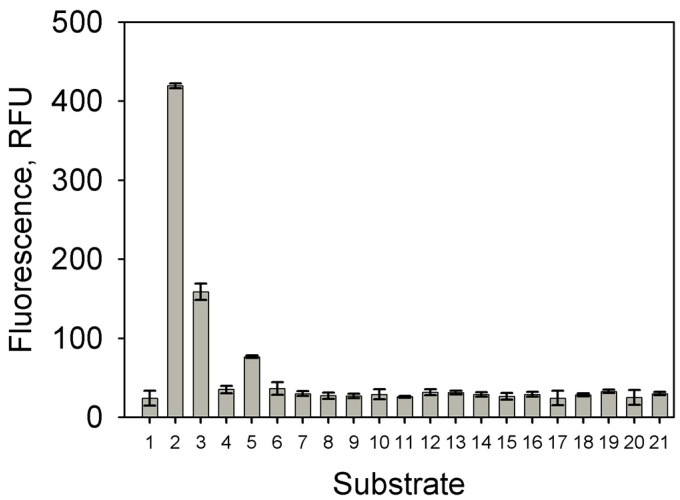
Substrate specificity of TpdR. 1—fluorescence of bacteria grown without inductor, 2—with tetramethylpyrazine, 3—2,3,5-trimethylpyrazine, 4—2,3-dimetylpyrazine, 5—2,5-dimetylpyrazine, 6—2,6-dimetylpyrazine, 7—2,3-diethyl-5-methylpyrazine, 8—3-methylpyridazine, 9—pyrazine, 10—2-pyrazinecarboxylic acid, 11—pyrazine-2,3-dicarboxylic acid, 12—5*H*-5-methyl-6,7-dihydrocyclopenta[*b*]pyrazine, 13—5,6,7,8-tetrahydroquinoxaline, 14—2,3,5-trimethylpyridine, 15—2,3-dimethylpyridine, 16—2,4-dimethylpyridine, 17—2,5-dimethylpyridine, 18—2,6-dimethylpyridine, 19—3,4-dimethylpyridine, and 20—3,5-dimethylpyridine, 21—pyridine. EGFP fluorescence was measured by a plate reader (λ_ex_ = 485 nm; λ_em_ = 510 nm); the data are presented as averages of the triplicate measurements with error bars.

To determine the substrate specificity of TpdR, the *R. jostii* TMP1 cells carrying the pART3-5′UTR-gfp plasmid were grown for 48 h in a NB medium supplemented with 5 mM of different pyrazines (2,3,5-trimethylpyrazine, 2,3-, 2,5-, 2,6-dimetylpyrazine, 2,3-diethyl-5-methylpyrazine, 3-methylpyridazine, pyrazine, 2-pyrazinecarboxylic acid, pyrazine-2,3-dicarboxylic acid, 5H-5-methyl-6,7-dihydrocyclopenta[b]pyrazine, and 5,6,7,8-tetrahydroquinoxaline) or pyridines (2,4,6-, 2,3,5-trimethylpyridine, 2,3-, 2,4-, 2,5-, 2,6-, 3,4-, 3,5-dimethylpyridine, and pyridine). The activity of the promoter was estimated by assaying the intensity of the EGFP fluorescence (Figure 7). The regulator was most sensitive to TTMP, but 2,3,5-trimethylpyrazine and 2,5-dimethylpyrazine induced the expression of EGFP as well. However, the values of the fluorescence intensity were almost three-fold and more than five-fold lower for 2,3,5-trimethylpyrazine and 2,5-dimetylpyrazine, respectively, compared with the result obtained for TTMP. No significant EGFP fluorescence was observed in the presence of the other aforementioned compounds.

## 3. Discussion

The minimal tetramethylpyrazine-inducible promoter P_TTMP_ is 138 nt long, and is located upstream of the putative flavin monooxygenase gene, *tpd*A, which is the first gene of the *Rhodococcus jostii* TMP1, *tpd*ABC operon. No homology was detected by comparing the P_TTMP_ promoter sequence with the known bacterial DNA sequences. This study shows that the activity of P_TTMP_ depends both on the concentration of TTMP and on the composition of the growth medium. The measurements of EGFP fluorescence intensity indicate that the highest level of expression is obtained when TTMP serves only as an inducer, but not as the sole source of carbon and energy. Both glucose and pyridine at low concentrations of 0.05 and 0.1% may be used as an additional carbon source for bacteria, because neither cause a catabolic repression of the P_TTMP_ promoter.

Based on the primer extension, transcription of the *tpd*ABC operon is likely regulated by two promoters, since two distinct transcriptional start sites, separated by six nucleotides, have been detected 45 and 52 nt upstream of the translation initiation codon of the *tpdA* gene. Notably, the transcription of several *Rhodococcus* spp. catabolic genes/operons described in the literature have been found to be initiated about 46–132 nt upstream of the coding region [10,28]. The predicted putative −35 (GGATTC and CATCCG) and −10 (TGCATT and TGAAGG) hexamers of both of the TTMP-depended promoters have been found to exhibit little sequence similarity to those of the other known *Rhodococcus* spp. promoters of catabolic genes, such as *thnA1*, *catA*, and *pheA2A1,* which encode mono- or di-oxygenases that are involved in the degradation of different aromatic compounds [6,7,29]. The P_TTMP_ promoter region contains two 15 bp direct repeats (box A and B) and two 7 bp inverted sequences (box C and D). It has been determined that the promoter lacking one of the two direct repeats, the box A motif, is inactive, suggesting that this sequence is essential for P_TTMP_ activity. It may be hypothesized that the sequence of box A is essential for the binding of the transcriptional regulator TpdR.

In 2011, Cappelletti and colleagues determined two overlapping imperfect inverted repeats of the conserved region upstream −35 motif of *alkB* promoter of *Rhodococcus* sp. BCP1 [5]. Two conserved inverted repeats were also found between −35 and −10 hexamers of the *tipA* promoter of *R. opacus* DSM44193 [10]. The inverted repeats and a potential hairpin structure was detected in the *dsz* promoter of *Rhodococcus erythropolis* IGTS8 [28]. In all the aforementioned studies, the inverted repeats were estimated to be the regulatory elements of the promoters. The promoter P_TTMP_, described in this study, contains two 7 bp long inverted repeats that form the stem of a putative hairpin structure. Presumably, this structure is important for DNA-hairpin-dependent promoter recognition by RNA polymerase and/or TpdR. As seen in Figure 5, the changes within the stem of the hairpin affect the activity of the P_TTMP_ promoter, likely due to the changes in a hairpin structure. Notably, a single point mutation T^‒33^ → A almost completely abolishes the transcription from P_TTMP_. We hypothesize that the latter substitution leads to the formation of the larger hairpin loop, which, in turn, likely prevents the binding of RNA polymerase and/or TpdR. We had been expecting that a five-nucleotide deletion (−34 to −30) in the stem region would result in a complete inactivation of the promoter. However, as seen in Figure 5, the level of the EGFP expression from the mutated promoter P_AS5_ only decreased by 50%, compared with that from P_TTMP_. This may be due to the formation of another, and perhaps less-stable, putative hairpin structure.

In this study, the transcriptional regulator TpdR, which is involved in the control of the TTMP degradation in the *R. jostti* TMP1 cells, has been identified. The protein belongs to the LAL subfamily (large ATP-binding regulators of the LuxR family) of transcriptional regulators, since it shares common structural features (size, *N*-terminal ATP-binding site containing Walker A and B motifs, and a *C*-terminal HTH motif) with the prototype of this family, the transcriptional activator MalT [26]. The LuxR family regulators generally act as activators and control a wide variety of functions in various biological processes, such as biofilm and spore formation, cell division, plasmid transfer, and bacterial virulence [30]. Up to now, the regulator DfdR from *Rhodococcus* sp. strain YK2 has been the only LuxR family protein described in rhodococci [9]. Both TpdR and DfdR contain a common *C*-terminal HTH motif, but the overall structure of these proteins is rather different. The protein TpdR has a P-loop NTPase domain in its *N*-terminus, whereas the DfdR contains no ATP-binding domains, yet has a GAF-like domain in the central part of the protein [9].

The TpdR regulator has a nucleotide-binding domain that contains both Walker A (nucleotide-binding) and Walker B (hydrolysis) motifs, suggesting that it is an AAA+ family protein. The AAA+ family proteins comprise the second major structural group of the larger P-loop protein superfamily [31]. The AAA+ proteins are functionally diverse and participate in many different cellular events, such as protein unfolding and degradation, DNA replication and repair, etc. Like all P-loop NTPases, AAA+ proteins have Walker A and Walker B motif residues that are critical for binding and hydrolyzing ATP [32]. Notably, only the Walker A motif has been detected in BpdS and AkbS regulatory proteins from *Rhodococcus* sp. M5 and DK17, respectively [33,34].

The LuxR family proteins interact with the target DNA via a HTH DNA-binding motif, and induce the transcription initiation by interacting with the aromatic substrate or with a pathway intermediate, which serves as an inducer molecule and provides regulatory specificity [3]. We suggest that, in the case of TpdR, both nitrogen atoms of the heterocyclic ring are essential for the interaction with the protein, since no promoter activity has been observed in the bacteria cultivated in the presence of pyridine derivatives. The P_TTMP_ promoter has been active only in the bacteria grown in the presence of 2,3,5,6-tetramethyl-, 2,3,5-trimethyl-, and 2,5-dimetylpyrazine, suggesting that the two methyl groups at the 2- and 5-positions of the pyrazine derivatives are crucial as well.

The regulators of the LuxR family contain a helix-turn-helix DNA binding domain and often bind to DNA sequences of dyad symmetry, located upstream of the target gene promoter [35]. For example, the transcriptional activators MalT from *Escherichia coli* and *Klebsiella pneumoniae* interact with two decanucleotide sequences, which are in a direct repeat [36], the regulatory protein NarL from *E. coli* binds to two heptamers arranged as an inverted repeat [37], whereas the transcription activator GerE from *Bacillus subtilis* binds two inverted 12 nt sequences [38]. Since, as seen in Figure 5, a nine-nucleotide deletion introduced into the region A completely abolished the activity of the promoter P_TTMP_ from *Rhodococcus jostii* TMP1, we suggest that the binding site for TpdR is a direct 15 nt-long repeat 5′-TnnAAnnGCGGAnTC-3′.

In conclusion, here, we present the results of the investigation of the transcriptional regulation of the *Rhodococcus jostii* TMP1 *tpdABC* operon, which is the first operon known to be involved in the biodegradation of tetramethylpyrazine in bacteria. The operon *tpdABC* contains three genes that are co-transcribed from the TTMP-inducible promoter P_TTMP_. To our knowledge, this is the only known promoter induced by TTMP, and it is the first characterized *Rhodococcus* sp promoter that is inducible by the *N*-heterocyclic compound. All previously described *Rhodococcus* spp. promoters have been induced by either aliphatic, aromatic, or *S*-, *O*-heterocyclic compounds [4,5,6,7,8,9,28,29]. In this study, a minimal P_TTMP_ promoter sequence and core promoter elements have been determined. Also, we show that the P_TTMP_ activity is regulated by the LuxR family transcriptional activato, TpdR. Taken together, the results presented here provide the basis for the development of novel expression vectors for the production of recombinant proteins in *Rhodococcus* spp. and, in addition, the TpdR protein is a promising scaffold for the design of an orthologous biosensor applicable in rhodococci.

## 4. Materials and Methods

### 4.1. Chemicals

Pyridine, 2,4,6-, 2,3,5-trimethylpyridine, 2,3-, 2,4-, 2,5-, 2,6-, 3,4-, 3,5-dimethylpyridine, 2,3,5-trimethylpyrazine, 2,3-, 2,5-, 2,6-dimetylpyrazine, 2,3-diethyl-5-methylpyrazine, 3-methylpyridazine, pyrazine, 2,3,5,6-tetramethylpyrazine, 2-pyrazinecarboxylic acid, pyrazine-2,3-dicarboxylic acid, 5*H*-5-methyl-6,7-dihydrocyclopenta[b]pyrazine, and 5,6,7,8-tetrahydroquinoxaline were purchased from either Sigma-Aldrich (Darmstadt, Germany) or Fluka (Berlin, Germany), and were of the highest purity available. The chemicals were used without additional purification. Nutrient agar, nutrient broth, and yeast extract were purchased from Oxoid (Hampshire, UK). The agar was from Merck (Darmstadt, Germany). The inorganic compounds were purchased from Lachema (Brno, Czech Republic). All compounds for DNA manipulation were purchased from Thermo Fisher Scientific (Vilnius, Lithuania).

### 4.2. Growth Media and Culture Conditions

All bacterial strains and plasmids used in this study are listed in Table 1. *Escherichia coli* were grown on a nutrient agar (NA) medium at 37 °C and in a nutrient broth (NB) medium at 30 °C aerobically. The *Rhodococcus jostii* TMP1 strain was cultivated in a NB, in mineral EFA medium (10 g/L K_2_HPO_4_, 4 g/L KH_2_PO_4_, 1 g/L (NH_4_)_2_SO_4_, 0.5 g/L yeast extract, 0.4 g/L MgSO_4_·7H_2_O, 10 mL/L salt solution [2 g/L CaCl_2_·2H_2_O, 1 g/L MnSO_4_·4H_2_O, 0.5 g/L FeSO_4_·7H_2_O, dissolved in 0.1 N HCl; pH 7.2]), or in minimal medium (5 g/L NaCl, 1 g/L K_2_HPO_4_, 1 g/L NH_4_H_2_PO_4_, 0.1 g/L MgSO_4_; pH 7.2) and on a NA, EFA, or minimal medium with agar (15 g/L) at 30 °C aerobically. The *R. erythropolis* SQ1 bacteria were grown on NA at 30 °C aerobically. The *E. coli* cells harbouring recombinant plasmids were grown in a NB medium, supplemented with 40 µg/mL of kanamycin. The *R. jostii* TMP1 and *R. erythropolis* SQ1 cells transformed with plasmids were grown in the presence of kanamycin (40 µg/mL and 60 µg/mL, respectively).

### 4.3. Genomic DNA Isolation

Genomic DNA from the *R. jostii* TMP1 bacteria was isolated using a method proposed by Woo et al. [41]. The plasmid DNA was purified using a phenol–chloroform extraction, precipitated by ethanol, and dissolved in distilled water. Standard techniques were used for further DNA manipulations [42].

### 4.4. PCR

*R. jostii* TMP1 genomic DNA was used for the PCR reactions. DNA fragments of different lengths, corresponding to the upstream region of the *tpdA* gene, were amplified using Maxima Hot Start PCR Master Mix (Thermo Fisher Scientific). The *tpdR* gene was amplified using a Long PCR Enzyme Mix (Thermo Fisher Scientific). The PCRs were performed according to the recommendations of the manufacturer, using Mastercycler ep gradient S (Eppendorf). The primers used in this study are listed in Table 2.

### 4.5. Preparation of the Electro-Competent Cells and Conditions of the Electroporation

*E. coli* competent cells were prepared by the method described by Sharma and Schimke [43]. *Rhodococcus* sp. competent cells were prepared following the method proposed by Gartemann and Eichenlaub [44]. DNA was mixed with 100 μL of ice-cold competent cells. Later, they were transferred to the electrocuvette (1 mm electrode gap, 100 μL capacity), and subjected to 20 kV/cm of electric pulse, with duration of 4.6‒5.6 ms. The pulsed cells were immediately diluted with 1 ml of a NB medium. The *E. coli* cells were incubated for 30‒45 min at 37 °C, whereas the *Rhodococcus* spp. Cells were incubated overnight at 30 °C. After the recovery, the cells were spread on agar plates containing kanamycin and/or appropriate substrates.

### 4.6. Enhanced GFP (EGFP) Fluorescence Measurement

To determine the optimal TTMP concentration for cell growth and catabolic repression, *R. jostii* cells harbouring pART3-5′UTR-gfp were grown in a medium supplemented with different TTMP concentrations and substrates (detailed in text). To study the effect of the P_TTMP_ promoter mutations, rhodococci harbouring a recombinant plasmid with a single mutation in the promoter region were grown in 20 ml of a NB medium, supplemented with 5 mM TTMP for 48 h. In all cases, the cells were collected by centrifugation (4000× *g*, 15 min. 10 °C) and prepared for the fluorescence measurements of cell suspension (OD_600_ 10), by the previously described method [24].

### 4.7. RNR Isolation and the Identification of the Transcription Start Site

The *R. jostii* TMP1 bacteria harbouring the pART3-5′UTR-gfp were cultivated in minimal medium containing 0.05% of TTMP, until the OD_600_ reached 0.5. In total, 1 mL of biomass was then collected by centrifugation (5 min, 16,100× *g*). The total RNA was isolated using ZR Soil/Fecal RNA MicroPrep kit (Zymo Research Corporation, Irvine, CA, USA). The 5′ end of the DNA primer (P_GFP_R), complementary to the EGFP gene sequence, was labelled with [γ-^32^P]-ATP (Amersham Biosciences, Cleveland, OH, USA), using T4 polynucleotide kinase (Thermo Fisher Scientific). Then, the primers were separated from the labelled nucleotides by precipitation with ethanol in the presence of 2 M of ammonium acetate. The primer extension analysis (Sanger et al., 1977) was performed on the total RNA extracted from *R. jostii* harbouring the pART3-5‘UTR-gfp, under conditions of primer excess, using the avian myeloblastosis virus (AMV) reverse transcriptase (Thermo Fisher Scientific), as described by Truncaite et al. [45]. The reaction products were analysed on a 6% denaturing polyacrylamide gel (8 M urea, TBE) and visualized using a Fujifilm FLA-5100 phosphorimager.

### 4.8. Mutagenesis of the P_TTMP_ Promoter Sequence

The site directed mutagenesis of the promoter sequence was carried out by the overlap extension method using a Phusion DNA Polymerase in a GC buffer with 3 mM MgCl_2_. The primers used to obtain the mutated promoter sequences are listed in Table 2. In the two-step PCR, the extension was 60 s at 60 °C temperature, and the other steps were performed according to the recommendations of the manufacturer, using the SensoQuest labcycler.

## Figures and Tables

**Table 1:** Bacterial strains and plasmids used in this study.

Strains	Description	Reference
*Escherichia coli* DH5α	ϕ80dlacZΔM15 Δ(lacZY-argF) U169 deoR recA1 endA1 hsdR17(r_K_^-^m_K_^+^) sup E44 thi-1 gyrA96 relA1	Thermo Fisher Scientific, Vilnius, Lithuania
*Rhodococcus jostii* TMP1	Utilise tetramethylpyrazine (TTMP) as a sole source of carbon and energy	[25]
*Rhodococcus erythropolis* SQ1		[39]
Plasmids		
pART3gfp	Km^R^; hybrid vector for nicotine-inducible enhanced GFP (EGFP) protein expression in *Arthrobacter* sp.; 6.1 kb	[40]
pART3-5′UTR-gfp	277 bp upstream *tpdA* gene (envolved in TTMP degradation) fragment was amplified and cloned to pART3gfp via *Bam*HI	[24]
pART3-5′UTR-gfp-R	*tpdR* gene (encoding transcription regulator) with upstream region (3 kb fragment) was amplified and cloned to pART3-5′UTR-gfp via *Xba*I	This work

**Table 2:** Primers used during this work.

Primers	Sequence 5′ → 3′	Purpose	Reference
5′UTR_F	TACGTGGATCCGTCAAGGAC	direct primer of the upstream *tpdA* region	[25]
UTR247	GATGGATCCGTGGTGGTCTTCGACC	determination of a minimal promoter sequence	This work
UTR227	GATGGATCCAGTGATGATGGTTCCGG	determination of a minimal promoter sequence	This work
UTR207	GATGGATCCTGGGTGCGTCCGACTC	determination of a minimal promoter sequence	This work
UTR187	GATGGATCCGCTGCAAAACGGAATC	determination of a minimal promoter sequence	This work
UTR157	GATGGATCCTCGGAGTTTGCGTACG	determination of a minimal promoter sequence	This work
UTR138	GATGGATCCATACGAAGCGACTTGAAAC	determination of a minimal promoter sequence	This work
UTR110	GATGGATCCAGTATCGGCTAGGTACA	determination of a minimal promoter sequence	This work
5′UTR_R	CACATGGATCCATCAAGATGAATCGC	reverse primer of the upstream *tpdA* region	[25]
Reg-F_Xba	GGATCTAGACCGAAGAACGAACG	*tpdR* amplification	This work
Reg-R_Xba	GTCTAGATCACAAACCAGTTCGC	*tpdR* amplification	This work
P_GFP_R	GGTGAACAGCTCCTCG	determination of transcription start site	This work
P-del-F	ACGAAGCGACTTGAATCAGTATCGGCTAG	P_TTMP_ mutagenesis	This work
P-del-atv	CTAGCCGATACTGATTCAAGTCGCTTCGT	P_TTMP_ mutagenesis	This work
P-TA-F	GGATTCCCAACCGTAGCCGAG	P_TTMP_ mutagenesis	This work
P-TA-atv	CTCGGCTACGGTTGGGAATCC	P_TTMP_ mutagenesis	This work
P-CA1-F	GGATTCCCATACGTAGCCGAGC	P_TTMP_ mutagenesis	This work
P-CA1-atv	GCTCGGCTACGTATGGGAATCC	P_TTMP_ mutagenesis	This work
P-CA2-F	GGATTCCCATCAGTAGCCGAGC	P_TTMP_ mutagenesis	This work
P-CA2-atv	GCTCGGCTACTGATGGGAATCC	P_TTMP_ mutagenesis	This work
P-GA-F	GGATTCCCATCCATAGCCGAGC	P_TTMP_ mutagenesis	This work
P-GA-atv	GCTCGGCTATGGATGGGAATCC	P_TTMP_ mutagenesis	This work
P-AS5-F	GGATTCCCGAAAATAGCCGAGC	P_TTMP_ mutagenesis	This work
P-AS5-atv	GCTCGGCTATTTTCGGGAATCC	P_TTMP_ mutagenesis	This work
